# Interactive Verification Analysis of Multiple Sequencing Data for Identifying Potential Biomarker of Lung Adenocarcinoma

**DOI:** 10.1155/2020/8931419

**Published:** 2020-10-01

**Authors:** Hongjun Fei, Songchang Chen, Chenming Xu

**Affiliations:** Department of Reproductive Genetics, International Peace Maternity and Child Health Hospital, Shanghai Key Laboratory of Embryo Original Diseases, Shanghai Municipal Key Clinical Specialty, Shanghai Jiao Tong University School of Medicine, Shanghai 200030, China

## Abstract

**Background:**

Lung adenocarcinoma (LUAD) comprises around 40% of all lung cancers, and in about 70% of patients, it has spread locally or systemically when first detected leading to a worse prognosis.

**Methods:**

We filtered out differentially expressed genes (DEGs) based on the RNA sequencing data in the Gene Expression Omnibus database and verified and deeply analyzed screened DEGs using a combined bioinformatics approach.

**Results:**

Expressions of 11,143 genes in 694 nontumor lung tissues and LUAD cases from 8 independent laboratories were analyzed; 188 mRNAs were identified as differentially expressed genes (DEGs). A PPI network constructed with 188 DEGs screened out 8 hub DEGs (*CDH5*, *PECAM1*, *VWF*, *CLDN5*, *COL1A1*, *MMP9*, *SPP1*, and *IL6*) which highly interconnected with other nodes. The expression levels of 8 hub genes in LUAD and control were assessed in the Oncomine database, and the results were consistent. The survival curves of 8 hub genes showed that their expressions are significantly related to the prognosis of lung cancer and LUAD patients except for *IL6*. Since the expression of *IL6* is nonspecific and highly sensitive, we choose the other 7 hub genes we had verified to do the next analysis. Mutual exclusivity or cooccurrence analysis of 7 hub genes identified a tendency towards cooccurrence between *CDH5*, *PECAM1*, and *VWF* in LUAD. The coexpression profiles of *CDH5* in LUAD were identified, and we found that *PECAM1* and *VWF* coexpressed with CDH5. Immunohistochemistry and RT-PCR analysis showed that higher levels of *CDH5*, *PECAM1*, and *VWF* were expressed in normal lung tissues but a low or undetectable level was found in LUAD tissues.

**Conclusions:**

Taken together, we speculate that *CDH5*, *PECAM1*, and *VWF* played an important role in LUAD.

## 1. Background

Lung cancers (LC) account for 13% of all cases in 2019 in the United States, and the greatest number of deaths are from LC whether in men (account for 24%) or women (account for 23%). One-quarter of all cancer deaths are due to LC which made it the leading cause of cancer-related mortality [1]. LC are mainly divided into two subtypes: non-small-cell lung carcinoma (NSCLC) and small-cell lung carcinoma (SCLC), accounting for 85% and 15% of all cases, respectively [2–4]. NSCLC can be classified into the major subtypes adenocarcinoma (AD) and squamous cell carcinoma (SCC). Lung adenocarcinoma (LUAD) is the most frequent histologic type of LC which comprises around 40% of all LC [5–7].

Patients whose LC have spread locally or systemically when first detected, constituting 70% of all patients, usually receive chemotherapy and/or radiation therapy instead of surgery [8–10]. Hence, local extension and metastases are also primary causes of death in LUAD patients. In the meantime and even more ominously, the recurrence rates in resected stage I NSCLC still range between 22% and 38% [11, 12], and nearly half of LUAD patients suffered a relapse and would die as a result of disease recurrence [13].

LUAD patients' risk assessment and therapeutic plan determination were usually dependent on traditional risk factors including tumor size, stage, and lymph node status. However, these existing clinical methods for prognosis evaluation still have defects such as invasiveness, unsystematic, and subjective; they cannot offer help for an effective targeted therapy and even do not clearly distinguish between patients who have a high or a low risk [14]. Therefore, it is necessary to establish a more accurate method to manage this high-mortality disease. It is urgent to find one or a few accurate indicators in the genesis and development of LUAD. We hope to shed light on exploring potential diagnostic and therapeutic targets in LUAD by our results of data analysis.

Although there are many studies about the mechanism of LUAD, the definite molecular cause of LUAD is still unclear. It is extremely vital and sorely demanded to reveal the pathogenesis and underlying molecular mechanisms of LUAD; it is beneficial for early diagnosis, prevention, and targeted therapy molecular biomarkers. In the present study, we want to find one or several molecular biomarkers which may eventually be applied to effective diagnosis and therapy of LUAD.

Microarray was a high-throughput platform which could measure the expression of the global gene. It was widely used for searching for possible genetic or epigenetic alternations, identifying molecular biomarkers such as for carcinomas [15, 16]. Huge amounts of core slice data were produced with extensive use of microarrays, and most of them were stored and shared in public databases [17, 18]. However, because of the limitations of some of these studies which included small study populations, single-center cohorts, and model overfitting, different researchers sometimes reached different conclusions. For getting more accurate reasons about onset and progression of LUAD, we integrated, reanalyzed, and verified the data stored in public databases. Some studies had been done to seek differentially expressed genes (DEGs) in LUAD though gene expression profiling microarrays [19–21]. However, for independent researches involving heterogeneous tissues or samples, in addition, their results were obtained from single cohort study, so their conclusions were limited or inconsistent. Consequently, key genes and pathways were difficult to confirm according to different studies. With our study, via integrating, reanalyzing, and verifying available and relevant expression profiling microarray datasets that have been uploaded in the Gene Expression Omnibus (GEO) database by different laboratories, one-sidedness of individual researches is overcome and statistical power increased; therefore, the screening results are more precise and reliable.

In the present study, we have downloaded 8 original microarray datasets, GSE32863 (58 nontumor lung tissues, 58 LUAD tissues), GSE7670 (28 nontumor lung tissues, 28 LUAD tissues), GSE40791 (100 nontumor lung tissues, 94 LUAD tissues), GSE63459 (32 nontumor lung tissues, 33 LUAD tissues), GSE75037 (83 nontumor lung tissues, 83 LUAD tissues), GSE85841 (8 nontumor lung tissues, 8 LUAD tissues), GSE116959 (11 nontumor lung tissues, 57 LUAD tissues), and GSE118370 (6 nontumor lung tissues, 6 LUAD tissues), from the GEO database (https://www.ncbi.nlm.nih.gov/geo). There were a total of 326 nontumor lung tissues and 367 LUAD tissues available. Subsequently, the DEGs were screened using R language, and 188 DEGs were filtered out from 11,143 genes based on 8 independent datasets which contained 694 cases. To better clarify the pathological mechanisms of LUAD, we performed cluster analysis, functional analysis, and biological pathway and process enrichment analysis for 188 screened DEGs. To determine hub genes with significant expression difference between normal lung and LUAD, we constructed a protein-protein interaction (PPI) network for the 188 DEGs screened with the threshold of ∣log_2_FoldChange | >2.0, and 8 hub genes were screened out. They are *CDH5*, *PECAM1*, *VWF*, *CLDN5*, *COL1A1*, *MMP9*, *SPP1*, and *IL6*. To verify our screening results, the expression signatures of hub DEGs in clinical cancer tissue were assessed by several databases. Their expressions in normal lung and LUAD tissues were analyzed in the Oncomine database. The survival times of normal and LUAD patients with high or low DEG expressions were identified with the KM Plotter database. The coexpression analysis of hub DEGs which was conducted by cBioPortal reveals the cooccurrence or mutual exclusivity relationship and provided the information for the possible underlying mechanism. All in all, we hope to gain further insight of LUAD at the molecular level and explored the potential candidate biomarkers for diagnosis, prognosis, and drug targets.

## 2. Materials and Methods

### 2.1. Microarray Data Selection

**Figure 1:**
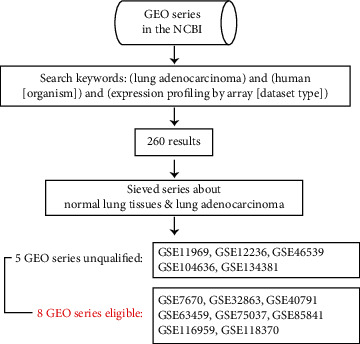
Process of screening and pooling 8 microarray gene expression datasets.

In the current study, the gene expression profiling datasets (ID: GSE32863, GSE7670, GSE40791, GSE63459, GSE75037, GSE85841, GSE116959, and GSE118370) were obtained from the Gene Expression Omnibus database of the National Center for Biotechnology Information (NCBI). “Lung adenocarcinoma,” “Homo sapiens [organism],” and “expression profiling by array [dataset type]” were used as keywords for searching. There were 260 results under this search condition. We selected the microarray datasets according to the following rules: the samples must contain LUAD tissues and normal lung tissues, no special treatment on patients, sequenced gene expression value > 0, and sequenced genes > 10,000. Under these conditions, we obtained 8 datasets to perform further analysis. We extracted expression data of all sequenced genes from the original studies by 8 independent researchers. The following information was extracted from each screened study: GEO accession number, sample type, platform, number of normal and LUAD tissues, and gene expression data. The information of the selected GEO series is listed in Table 1. We download the raw data of 693 specimens from 8 independent GEO series. In total, 326 nontumor tissues and 367 LUAD specimens were enrolled in 8 GEO series. The process of data filing is shown in Figure 1.

### 2.2. Data Preprocessing before Difference Analysis

We utilized the robust multiarray average algorithm of the Affy package in R language to convert the raw data to expression data. According to the platform annotation files, the expression levels of the probe sets were converted into gene expression levels by the Bioconductor annotation function of R. Expression values of multiple probes for a given gene were averaged. With this, we obtained 8 tables containing expression value of tested genes based on 8 GEO series. Then, we make use of the sameGene package in R to merge the gene expression data of 693 patients from datasets of GSE32863, GSE7670, GSE40791, GSE63459, GSE75037, GSE85841, GSE116959, and GSE118370 into one output table according to the same gene names. Then, the datasets of the output table were assigned into 2 groups: normal lung group and LUAD group. Batch normalization was conducted on all expression profiling data using the ComBat algorithm in the Surrogate Variable Analysis package of R language. The normalization can eliminate the systematic variations among different studies.

### 2.3. Differentially Expressed Gene (DEG) Screening

The DEGs were selected from the normalized data of normal lung and LUAD tissues using the linear models for microarray data (Limma) package in Bioconductor (http://www.bioconductor.org/packages/release/bioc/html/limma.html). The filter criteria is ∣log_2_FoldChange |  (∣log_2_FC∣) > 2, also known as ∣FoldChange | >4 and adjusted *p* value < 0.05.

A volcano plot, representing the distribution of the fold change and *p* value of all genes, was drawn. A heat map of expression hierarchical clustering analysis for 188 DEGs was performed to investigate probable discrepancies between normal lung and LUAD tissues.

### 2.4. Functional and Pathway Enrichment Analysis for All DEGs

To explore the main molecular function and pathway that involved DEGs, we did functional enrichment analysis using FunRich. The FunRich software is a standalone functional enrichment and network analysis tool. It was utilized to perform cellular component, functional (molecular function and biological process) and pathway (biological pathway) enrichment analysis for the obtained DEGs with *p* value < 0.05 as a strict cutoff.

### 2.5. Protein-Protein Interaction (PPI) Network Construction and Hub Gene Identification

The functional protein-protein interaction (PPI) analysis is essential to interpret the molecular mechanisms of key cellular activities in carcinogenesis. It is constructed on the basis of the Search Tool for the Retrieval of Interacting Genes (STRING) database [22]. Our study constructed a PPI network for all DEGs and visualized the interaction network with the cutoff criterion of interaction score > 0.4.

Hub genes were selected with interaction degree > 15, and finally, there are 8 hub genes we selected which were highly interconnected with other nodes.

### 2.6. Oncomine Database Analysis and Kaplan-Meier Plotter Analysis for DEGs

Oncomine is a cancer transcriptomic database and web-based discovery platform with genome-wide expression analyses of various cancers [23, 24]. The expression levels of 8 screened hub DEGs were analyzed using the Oncomine Cancer Profiling Database (https://www.oncomine.org). We analyzed and compared the expression of 8 screened hub genes between LUAD tissues and normal lung tissues in the Oncomine database.

The Kaplan-Meier Plotter is a database that can be used to assess the effect of 54,675 genes on patient survival using 10,461 cancer samples (breast, ovarian, lung, and gastric cancer) [25]. For survival analyses, we analyzed the prognostic value of 8 screened hub DEGs in normal lung and LUAD using the Kaplan-Meier Plotter (http://kmplot.com/analysis/) and tested for significance using logrank tests. The analysis was performed according to the manufacturer's instructions. ALL hub DEGs had a significant correlation with the overall survival of LC and LUAD patients except IL6.

Coexpression analysis in Oncomine was used to identify sets of genes with synchronous expression patterns. The coexpression profiles of *CDH5* in LUAD was identified and presented as the pattern of heat map.

### 2.7. Genetic Alteration and Coexpression Analysis of Screened Hub DEGs

The cBioPortal (http://www.cbioportal.org) is an open-access resource for interactive exploration of multidimensional cancer genomics datasets [26]. We studied alterations (amplification, deep deletion, missense mutation, inframe mutation, truncating mutation, mRNA upregulation, and mRNA downregulation) in *VWF*, *CLDN5*, *CDH5*, *COL1A1*, *MMP9*, *PECAM1*, and *SPP1* genes in LUAD (TCGA, provisional) case set using cBioPortal. The cBioPortal is also used for cooccurrence or mutual exclusivity and customizable correlation analysis.

## 3. Quantitative Real-Time PCR Analysis and Immunohistochemistry

Total RNA in 2 paired lung and LUAD tissues was extracted using TRIzol (Invitrogen). The cDNA was reverse-transcripted from 1 mg of total RNA using the reverse transcriptase kit (Toyobo). Q-PCR was performed using a 7500 Real-Time PCR System (Applied Biosystems) and SYBR Green PCR Master Mix (BioRad). GAPDH was used for normalization. Primers sequences were listed in Table 2.

## 4. Results

### 4.1. Normalization of Gene Expression Data

**Figure 2:**
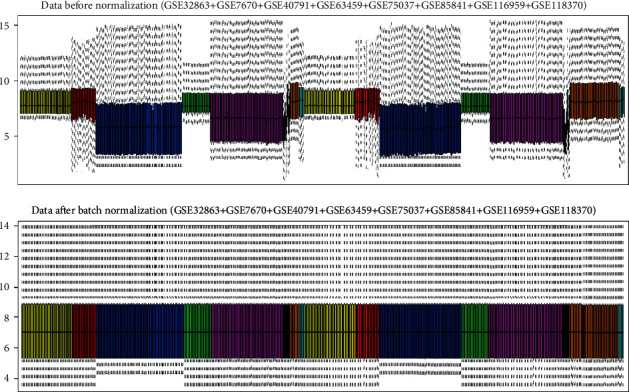
Box figures of expression values of all genes before and after normalization. The results before and after normalization shown in the top and bottom box plots, respectively, describe the expression values of 694 samples from GSE32863, GSE7670, GSE40791, GSE63459, GSE75037, GSE85841, GSE116959, and GSE118370 datasets. The same-colored columns represent the samples from the same GEO series. The 8 groups (in order with different colors) on the left were normal lung tissues and the 8 groups (in order with different colors) on the right were LUAD tissues.

Expression data of 11,143 genes from 693 samples (326 nontumor lung tissues and 367 LUAD specimens) were normalized with the median method following batch normalization. The expression values of all specimens before and after normalization are shown in the top and bottom box figures in Figure 2. The horizontal axis stands for different samples.

The vertical axis stands for gene expression value. The black horizontal line represents the median of expression value of the sample, which is almost on a straight line after batch normalization, suggesting that normalized data were qualified.

### 4.2. Selection of DEGs and Expression Hierarchical Clustering Analysis

We used R Limma package software to analyze which gene sets were aberrantly expressed in comparisons with the threshold of ∣log_2_FC | >2 and *p* < 0.05. The DEGs were identified using *t* test statistical algorithm. The significant genes' lists were selected according to fold change of gene expression values.

**Figure 3:**
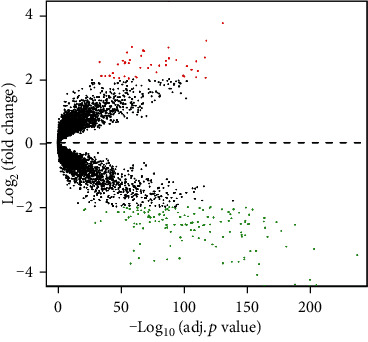
Volcano plot of the aberrantly expressed genes. The red spots represent upregulated genes in which ∣Log_2_FoldChange | >2.0; the green spots represent downregulated genes in which ∣Log_2_FoldChange | >2.0. Black spots show the genes with expression of ∣Log_2_FoldChange | ≤2.0.

**Figure 4:**
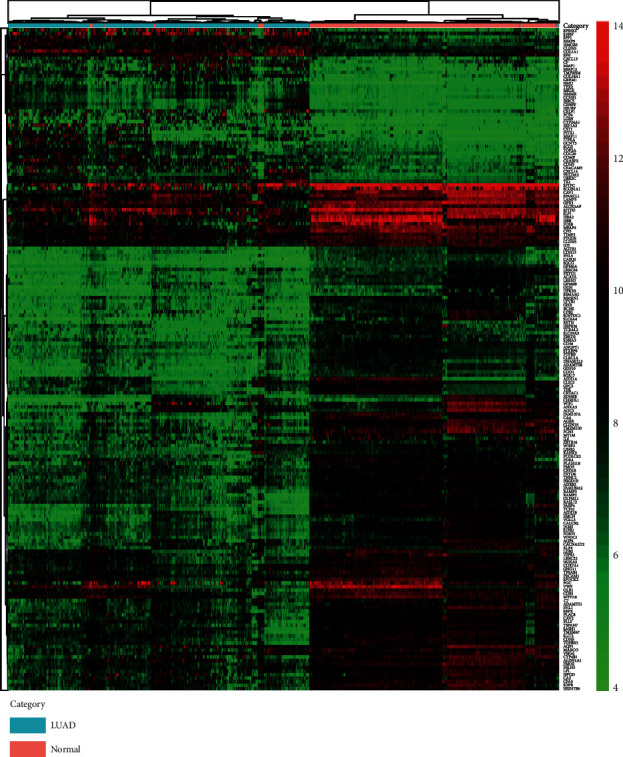
Heat map of expression hierarchical clustering analysis for 188 DEGs filtered from 694 specimens.

In total, 188 DEGs (44 upregulated and 144 downregulated) were obtained based on the gene expression data of 693 patients (326 normal lung and 367 LUAD specimens from 8 GEO series). We list the top 40 DEGs according to the fold change of the gene expression value in Table 3. The volcano plot (Figure 3) showed the distribution of all DEGs. The volcano plot shows the distributions of fold change [(log_2_FoldChange] (*Y*-axis) and *p* values [-log_10_ (*p* value)] (*X*-axis). In Figure 4, fold change patterns of all DEGs were selected, analyzed, and displayed in a heat map to evaluate and compare differences in gene expression between normal lung and LUAD.

### 4.3. Function and Pathway Enrichment Analysis of all DEGs

**Figure 5:**
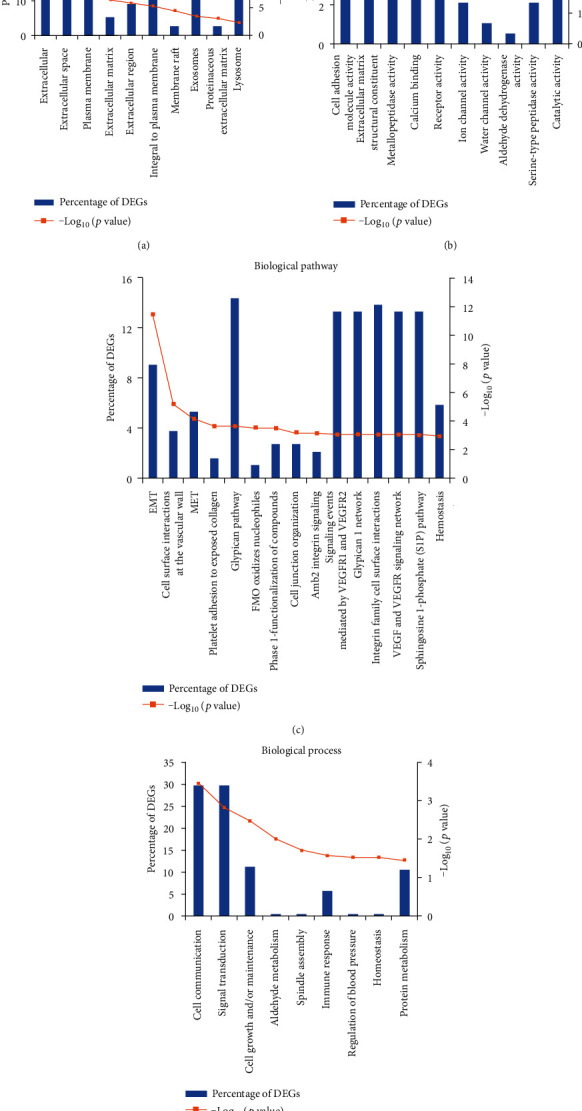
Cellular component (a), molecular function (b), significant biological pathway (c), and biological process (d) enrichment analyses of 188 differentially expressed genes (DEGs). The *Y*-axis represents the percentage of DEGs and -log_10_(*p* value); the *X*-axis represents enriched cellular components, molecular functions, biological processes, and pathways.

Cellular component enrichment analysis of all DEGs described their distribution and structure (Figure 5(a)). About the molecular function, the DEGs significantly enriched in cell adhesion molecule activity, extracellular matrix structural constituent, metallopeptidase activity, calcium ion binding, and receptor activity (Figure 5(b)). To better clarify the pathological mechanisms, we performed biological pathway enrichment analysis. According to the result of the pathway enrichment analysis, DEGs were mainly enriched in epithelial-to-mesenchymal transition (EMT), cell surface interactions at the vascular wall, mesenchymal-to-epithelial transition (MET), platelet adhesion to exposed collagen, and so on (Figure 5(c)).

To further investigate the biological effects of aberrantly expressed DEGs in LUAD, the biological process enrichment analysis of 188 screened DEGs was carried out. The top 9 enriched biological processes are shown in Figure 5(d). The functions in the biological process category were enriched in cell communication, signal transduction, cell growth and/or maintenance, aldehyde metabolism, and so on.

### 4.4. PPI Network Construction and Hub Gene Selection

**Figure 6:**
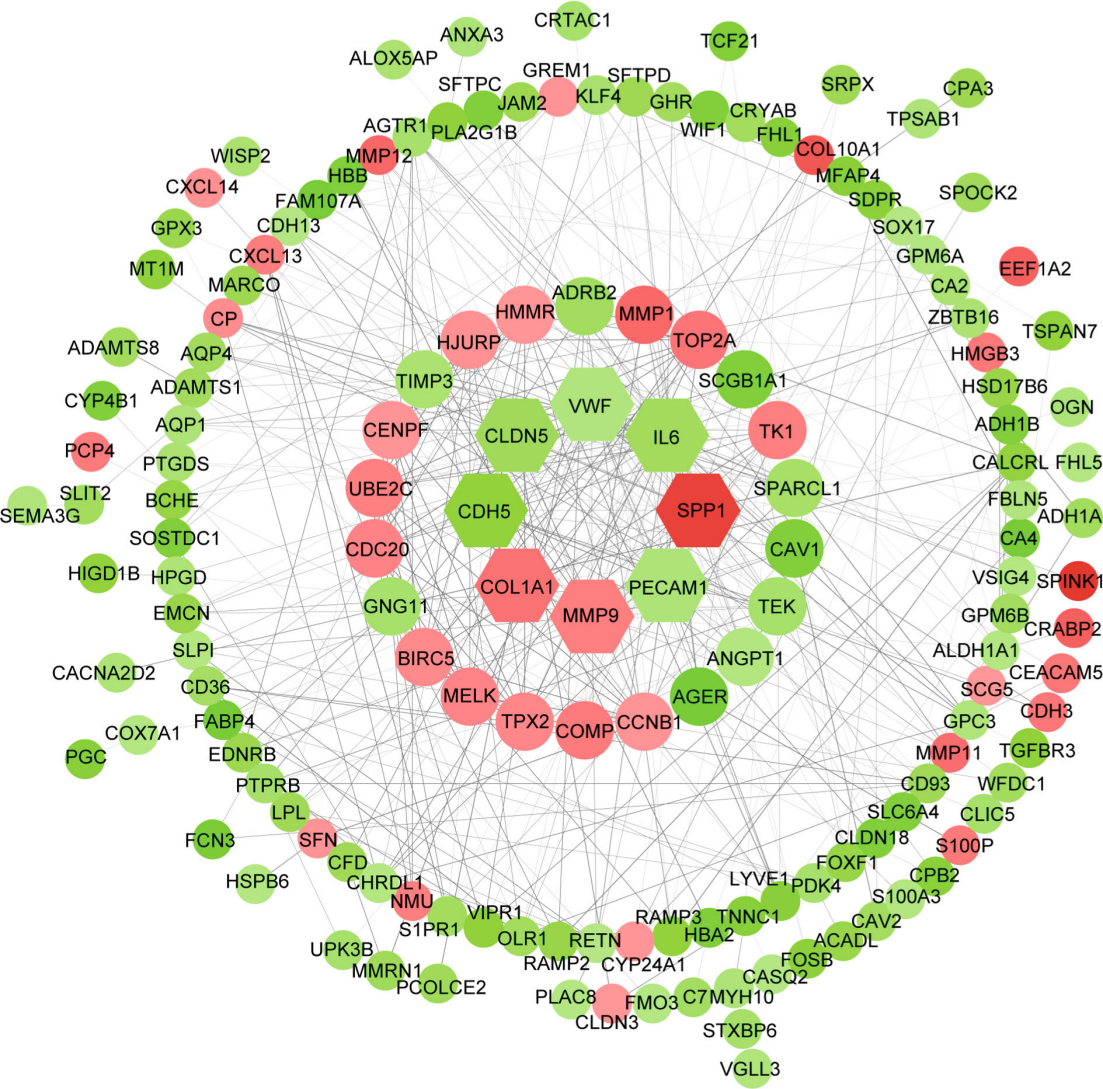
PPI network of 188 screened DEGs using ∣log_2_FoldChange | >2.0 as the screening index. The color of nodes is according to log_2_FoldChange: red nodes denote upregulated DEGs in which log_2_FoldChange > 0 and green nodes denote downregulated DEGs in which log_2_FoldChange < 0. The width of the edge has positive correlation with the combined score of protein interaction. The size of nodes is inversely related to *p* value. The innermost circle nodes (*CDH5*, *PECAM1*, *VWF*, *CLDN5*, *COL1A1*, *MMP9*, *SPP1*, and *IL6*) denote core DEGs also called hub genes in which interacting proteins > 15.

Based on the information in the STRING protein query from public databases, we constructed the PPI network for 188 DEGs using ∣log_2_FoldChange | >2.0 as the screening index (Figure 6); there are 8 hub genes selected with interaction degree > 15. They are shown in the innermost circle. The op 8 hub genes were *CDH5*, *PECAM1*, *VWF*, *CLDN5*, *COL1A1*, *MMP9*, *SPP1*, and *IL6*.

### 4.5. Validation of the Expression of Obtained Hub DEGs in Oncomine Database

**Figure 7:**
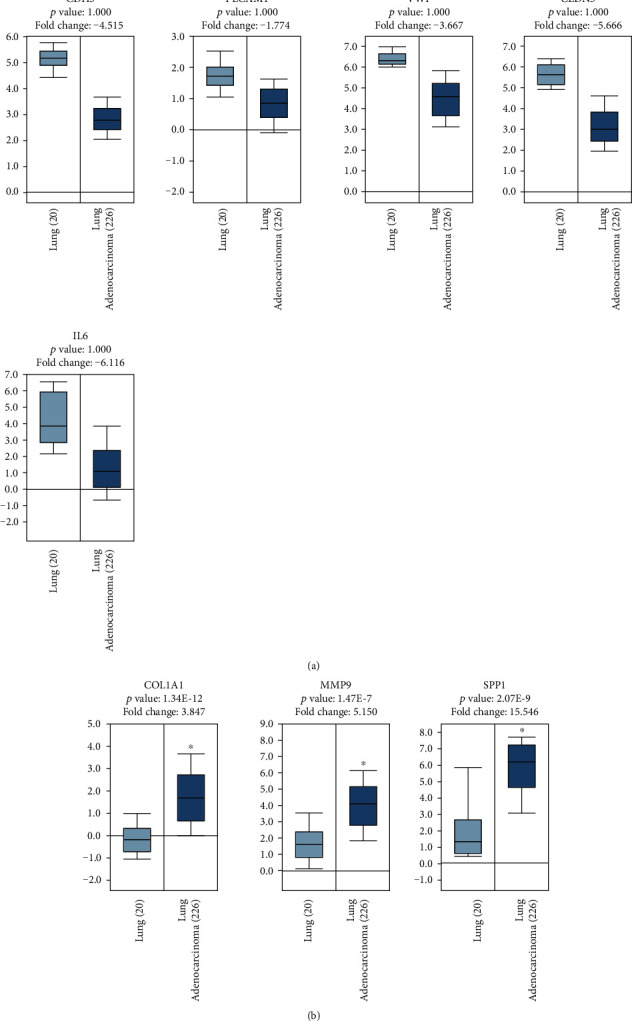
Comparing expressions of 8 hub genes (3 upregulated and 5 downregulated) between normal lung and LUAD tissues in the Oncomine database. Box plots derived from gene expression data in the Oncomine database comparing expression of the downregulated hub DEGs (a) and upregulated hub DEGs (b) in normal lung (light blue columns) and LUAD tissues (dark blue columns). The *X*-axis indicates tissue types. The *Y*-axis represents normalized expression of mRNAs.

To further elucidate whether the expressions of the DEGs in LUAD patients were consistent with our analysis result based on GEO data, a clinical study was performed in the light of previous results in cancer microarray database of Oncomine. The expressions of 8 hub DEGs were verified and are shown in Figure 7. There are 5 downregulated DEGs and 3 upregulated DEGs in LUAD. The expression trend of 8 DEGs is in accordance with our results obtained from the GEO sequenced data. The differences had statistical significance in upregulated DEGs (*p* < 0.001), but were not statistically significant in downregulated DEGs although the expression of DEGs had a trend of downregulation in LUAD.

### 4.6. Survival Analysis for Obtained Hub DEGs with Kaplan-Meier Plotter

**Figure 8:**
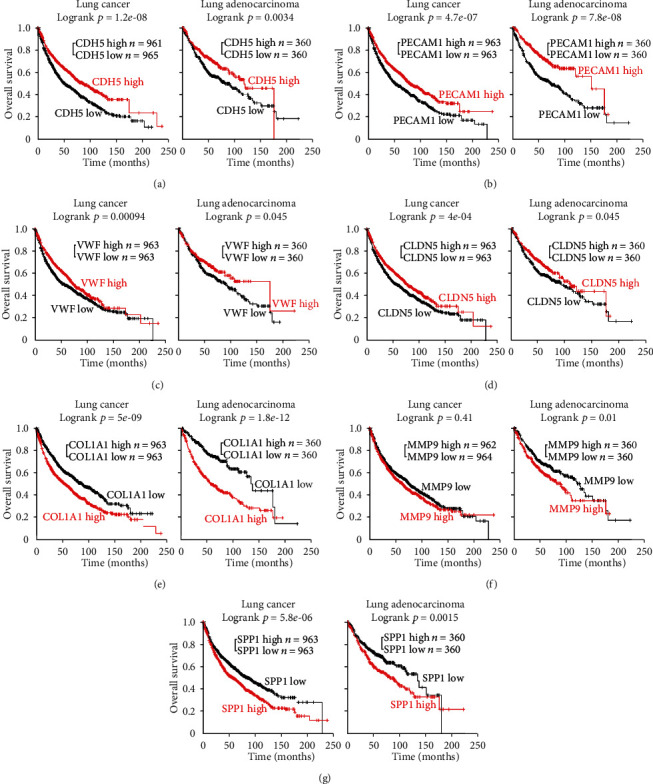
Prognostic value of 7 remaining hub genes except IL6 in lung cancer and LUAD. Data were obtained from the Kaplan-Meier Plotter database. The *p* value was calculated by a logrank test.

According to our previous bioinformatics analyses and validation, the hub genes' expression in LUAD patients in the Oncomine database is consistent with our research results from the GEO series. To explore the association of 8 hub gene expressions with the prognosis of LUAD patients, the survival curves were drawn using the Kaplan-Meier Plotter database. As show in Figure 8, the low expressions of *CDH5*, *PECAM1*, *VWF*, and *CLDN5* were associated with worse prognosis(*p* < 0.05), and the high expressions of *COL1A1*, *MMP9*, and *SPP1* were associated with worse prognosis (*p* < 0.05). The differences were statistically significant. In other words, LUAD patients with low expression of screened upregulated hub genes had a better prognosis, and low expression of screened downregulated hub genes had worse prognosis except for the IL6 gene. As a characteristic cytokine expressed in plasma and associated with inflammation, IL6's expression is nonspecific and sensitive, so we choose the other 7 hub genes we had verified to do the next analysis.

### 4.7. Coexpression Analysis and Genetic Alterations of Obtained Hub DEGs in LUAD

**Figure 9:**
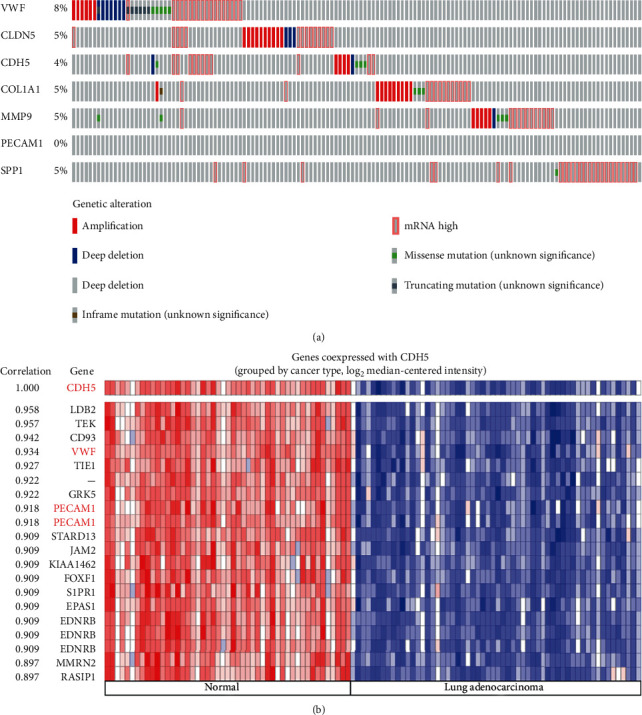
Genetic alteration analysis of screened 7 hub genes (a) and coexpression profile analysis of *CDH5* (b).

The OncoPrint from cBioPortal is a concise and compact graphical summary of genomic alterations in multiple genes across a set of tumor samples. It summarized distinct genomic alterations including mutations, CNAs (amplifications and homozygous deletions), and changes in gene expression or protein abundance. Based on previous results of difference analysis, expression validation, PPI networks construction, and survival analysis, *VWF*, *CLDN5*, *CDH5*, *COL1A1*, *MMP9*, *PECAM1*, and *SPP1* were hub genes highly interconnected with other DEGs. The expression of these hub genes was compared and analyzed in the GEO database and Oncomine database; their expression differences between normal lung and LUAD tissues were certain and obvious. And the relationship between their expressions and overall survival was validated; there is significant correlation between hub genes' expression and LUAD patients' survival time. We analyzed genomic alterations of the screened hub DEGs using cBioPortal and visualized gene alterations across a set of LUAD cases (Figure 9(a)). OncoPrint can also help identify trends such as mutual exclusivity or cooccurrence between genes. The mutual exclusivity and cooccurrence from cBioPortal can be exploited to identify previously unknown mechanisms that contribute to oncogenesis and cancer progression, so we used cBioPortal to explore the potential relationship between 7 hub genes. As Table 4 shows, there was a tendency towards cooccurrence between *CDH5* and *PECAM1* or *VWF* in LUAD (*p* < 0.05).

Coexpression analysis in Oncomine is a tool which can be used to identify sets of genes with synchronous expression patterns. The coexpression profiles of *CDH5* in LUAD were identified and presented as the pattern of the heat map. We identified the coexpression profiles for *CDH5* with a strong cluster of the top 20 genes across a panel of 107 LUAD tissues. The result showed that, as DEGs that we screened out from LUAD and control tissues based on the GEO database, *PECAM1* and *VWF* coexpressed with *CDH5* (Figure 9(b)).

### 4.8. Immunohistochemistry and RT-PCR Analysis of CDH5, PECAM1, and VWF

**Figure 10:**
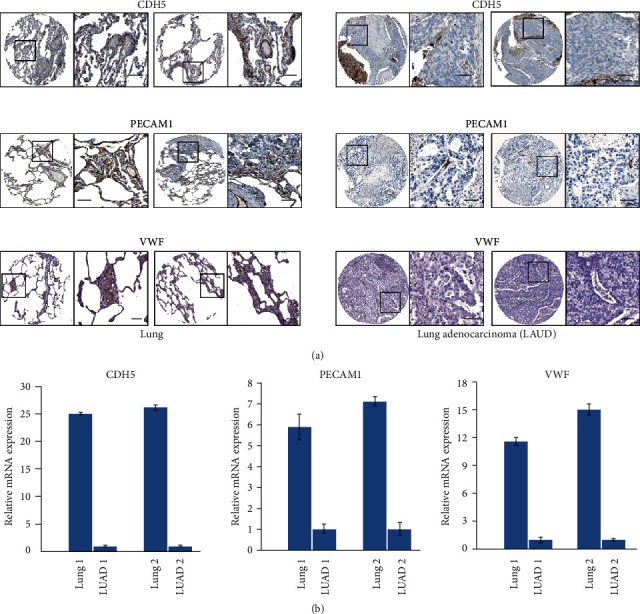
Immunohistochemistry and RT-PCR analysis of *CDH5, PECAM1* and *VWF*. (a) *CDH5, PECAM1* and *VWF* protein were detected by immunohistochemistry in lung tissues (left panel) and LUAD tissues (right panel). Pictures were adopted from the Human Protein Atlas (http://www.proteinatlas.org). Scale bar, 100 *μ*m. (b) *CDH5, PECAM1* and *VWF* mRNA expression by real-time PCR analysis in 2 paired lung and LUAD tissues.

According to our results, *CDH5*, *PECAM1*, and *VWF* were DEGs we screened with the threshold of ∣log_2_FC | >2.0 and *p* < 0.05, and they were hub genes in the PPI network; their expression in normal lung and LUAD was verified in the Oncomine database, and their expressions were significantly related to the prognosis of lung cancer and LUAD patients; moreover, there is a tendency towards cooccurrence between *CDH5*, *PECAM1*, and *VWF* in LUAD. We further verified the expression of *CDH5*, *PECAM1*, and *VWF* through immunohistochemistry and RT-PCR analysis; immunohistochemistry (IHC) data from the Human Protein Atlas (http://www.proteinatlas.org) indicated strong expression of *CDH5*, *PECAM1*, and *VWF* protein in lung tissues, but not in LUAD tissues (Figure 10(a)). The mRNA levels of *CDH5*, *PECAM1*, and *VWF* were noticeably decreased in LUAD tissues compared to paired lung tissues (Figure 10(b)).

## 5. Conclusion

Our study utilized analysis of whole genome sequencing results from different laboratories and screened out DEGs from 5 different sequencing platforms containing 8 original microarray datasets and 694 cases. There were 44 upregulated DEGs and 144 downregulated DEGs in LUAD with the threshold of ∣log_2_FC | >2.0 and *p* < 0.05. Biological process analysis, biological pathway analysis, and PPI network analyses provided a set of related genes and pathways to help elucidate the molecular mechanisms of LUAD. Validation experiments verified that the expression levels of DEGs in the Oncomine database are consistent with their expression levels in the GEO series. The survival curves of hub genes showed that the expressions of hub genes were significantly related to the prognosis of LUAD patients (*p* < 0.05) except for *IL6*. At this point, we believe *CDH5*, *PECAM1*, *VWF*, *CLDN5*, *COL1A1*, *MMP9*, and *SPP1* play a vital role in LUAD. Mutual exclusivity or cooccurrence analysis of screened 7 hub genes showed that there was a tendency towards cooccurrence between *CDH5*, *PECAM1*, and *VWF* in LUAD (*p* < 0.05). Then, the coexpression profiles for *CDH5* obtained based on Oncomine showed that *PECAM1* and *VWF* coexpressed with *CDH5* in LUAD, and they were also DEGs that were screened out from LUAD based on our previous results. Immunohistochemistry and RT-PCR analysis showed that higher levels of *CDH5*, *PECAM1*, and *VWF* were expressed in normal lung tissues but a low or undetectable level was found in LUAD tissues. From all above results, we speculate that *CDH5*, *PECAM1*, and *VWF* play an important role in LUAD. Though analyzed all GSE series-compared normal lung and LUAD tissues in the GEO database; the prediction is more accurate and bias of individual studies can be overcome. Our study provides information for researchers to identify possible candidate genes and pathways which may be involved in LUAD for further studies.

## 6. Discussion

Worldwide, approximately 2,093,800 patients are diagnosed with lung cancer each year, and 1,761,000 are expected to succumb to the disease in 2018. Statistically, in both sexes combined, lung cancer is the most commonly diagnosed cancer (11.6% of the total cases) and the leading cause of cancer death (18.4% of the total cancer deaths) [27, 28]. LC is the most frequent cancer and the leading cause of cancer death among males and women in 2019 in the United States [1]. There are 2 main forms of LC: NSCLC (85% of patients) and small-cell lung cancer (SCLC) (15%). Adenocarcinoma is the most common type of NSCLC and accounts for approximately 40% of lung cancers [29–31]. The most common diagnostic mean for LC is fiberoptic bronchoscopy; it can help to diagnose NSCLC, but quite often, the amount of obtained material is not sufficient to subclassify NSCLC in more detail or for targeted therapies [32]. The vast majority of LC patients are diagnosed until advanced-stage LC, so that they had a worse prognosis and a high risk of distant recurrence and death [33]. We know little about the target for early detection of LUAD. Consequently, there is an urgent need for diagnostic molecular features or biomarkers that can be associated with survival and disease recurrence in LUAD.

A field which has recently contributed significantly to improve diagnostics, classification, and prognostics is the LUAD transcriptomics microarray, a whole transcriptome high-throughput sequencing and analysis technique which identifies changes in the mRNA expression, and is now being used to gain a more detailed understanding of the molecular mechanism of LUAD [34, 35]. Employing analysis of whole transcriptome sequencing results from different laboratories, statistical power increased and prediction is more accurate; moreover, bias of individual studies can be overcoming. In the current study, we focused on the aberrantly expressed mRNAs in LUAD based on GEO RNA-seq data, and the common DEGs that were screened out from different researchers containing 693 samples were listed. There were 44 upregulated DEGs and 144 downregulated DEGs in LUAD with the threshold of ∣log_2_FC | >2 and *p* < 0.05.

Biological pathway analysis of all DEGs showed that the DEGs were mainly involved in epithelial-to-mesenchymal transition (EMT), cell surface interactions at the vascular wall, mesenchymal-to-epithelial transition (MET), platelet adhesion to exposed collagen, and glypican pathway. In the past decades, an increased number of studies have shown that EMT is associated with poor prognosis in different tumor types including NSCLC [36, 37]. EMT, as well as its reverse process, MET, is thought to be involved in the pathogenesis of numerous lung diseases ranging from developmental disorders and fibrotic tissue remodelling to lung cancer [38]. Kakolyris et al. have shown previously in NSCLC an association between high mitogenic/angiogenic factor expression with high angiogenesis and poor prognosis [39]. Glypican-3 (GPC3) is a membrane-bound proteoglycan, belonging to the glypican-related integral membrane proteoglycan family, which includes six members (GPC1–GPC6). It has been identified as a potential biomarker candidate in lung carcinoma, severe pneumonia, and acute respiratory distress syndrome (ARDS) [40]. Glypican-5 (GPC5) was a novel tumor metastasis suppressor in LUAD through suppresses EMT [41]. Function analysis can help us better understand the mechanism of LUAD and provide guidance for LUAD prevention and treatment; however, further laboratory and clinical researches are required.

The PPI network of 188 DEGs which were screened from 693 LUAD and control tissues using ∣log_2_FoldChange | >2.0 as the screening index helped us find 8 hub DEGs which had the most functional connections: *CDH5*, *PECAM1*, *VWF*, *CLDN5*, *COL1A1*, *MMP9*, *SPP1*, and *IL6*. The 8 hub genes interact with a protein number at least >15. To verify our previous results in this paper, we assessed the expression levels of the 8 hub DEGs. The expression levels of the 8 hub DEGs were analyzed in the Oncomine database, respectively. The expression trend of 8 DEGs is in accordance with our results obtained from the GEO sequenced data. To verify our results, we analyzed further the relationship between hub genes' expression and prognosis. The hub genes that we screened as upregulated in LUAD were correlated with poor prognosis, and the hub genes that we screened as downregulated in LUAD were associated with favorable prognosis except for IL6. From all the above results, considering candidate biomarkers' characteristics of relative stability, further analysis was performed with the remaining 7 hub genes except IL6.

We draw survival curves of the screened hub genes and found that the prognoses of LC/LUAD patients were statistically significant with hub genes' expression (*p* < 0.01). OncoPrint helped us identify trends such as mutual exclusivity or cooccurrence of screened hub genes. We found that there was a tendency towards cooccurrence between *CDH5*, *PECAM1*, and *VWF* in LUAD (*p* < 0.05). Then, coexpression analysis with the Oncomine database for *CDH5* found that *CDH5* coexpressed with *PECAM1* and *VWF* in LUAD, and they were also DEGs that were screened out from LUAD based on our previous results. Our results seem to show that *CDH5*, *PECAM1*, and *VWF* play a vital role in LUAD. *CDH5* encodes Cadherin-5, which is localized at intercellular junctions of endothelial cells and plays an important role in the control of vascular integrity and permeability, and contributes to endothelial cell assembly in tubular structure [42]. Many studies had reported that *CDH5* expression is associated with multiple tumors [43, 44], such as gastric cancer and breast cancer, but the relationship between *CDH5* and LUAD is still to be determined. *PECAM1* is a multifunctional cell adhesion molecule involved in numerous physiologic processes within the vasculature; Abraham et al. found that the activity of *PECAM1* appears to be associated with the tumor microenvironment and tumor cell proliferation [45]; Kuang et al. demonstrated that *PECAM1* could be a potential prognostic factor and therapeutic target in NSCLC [46]. The von Willebrand factor (VWF) is a multimeric glycoprotein and plays an essential role in mediating platelet-tumor cell interactions [47]. The relationship between *VWF* and LUAD is still underway. From all the above results, we speculate that *CDH5*, *PECAM1*, and *VWF* play an important role in LUAD.

This study had several limitations. Firstly, the expression of screened downregulated hub genes in LUAD patients in the Oncomine database was not statistically significant (*p* > 0.05), but based on the figure, the trends of hub genes' expression were consistent with the GEO database; statistical nonsense may be because of insufficient samples. Second, even though we performed preliminary validation of the results, more in-depth studies are needed in the future. Therefore, we hope that these results can be integrated into future experiments and facilitate further understanding of the molecular mechanisms of LUAD.

Despite these limitations, we believe that this analysis represents a valuable resource and can be considered as a preliminary study for future studies of LUAD. Our study provides information for researchers to identify possible candidate genes and pathways which may be involved in LUAD for further studies. We gained further insight of LUAD carcinogenesis at the molecular level and explored the potential candidate biomarkers for diagnosis, prognosis, and drug targets.

## Figures and Tables

**Table 1:** Characteristic of included microarray data.

Expression profiling array (normal & LUAD)	Platforms	GEO accession	Samples
Genome	GPL570	GSE40791	100 normal; 94 LUAD
		GSE118370	6 normal; 6 LUAD
	GPL6884	GSE32863	58 normal; 58 LUAD
		GSE63459	32 normal; 33 LUAD
		GSE75037	83 normal; 83 LUAD
	GPL96	GSE7670	28 normal; 28 LUAD
	GPL20115-26806	GSE85841	8 normal; 8 LUAD
	GPL17077-17467	GSE116959	11 normal; 57 LUAD

**Table 2:** .

CDH5	5′-TACCAGGACGCTTTCACCAT-3′
	5′-AAAGGCTGCTGGAAAATGGG-3′

PECAM1	5′-GCATATCCAAGGTCAGCAGC-3′
	5′-TCTGGATGGTGAAGTTGGCT-3′

VWF	5′-CCTTGAATCCCAGTGACCCT-3′
	5′-ACTTCAAACTCAGCCTCGGA-3′

GAPDH	5′-CTCCTCCTGTTCGACAGTCAGC-3′
	5′-CCCAATACGACCAAATCCGTT-3′

Immunohistochemistry of *CDH5*, *PECAM1*, and *GAPDH* on lung and LUAD tissue was adopted from the Human Protein Atlas (http://www.proteinatlas.org).

**(a):** 

Upregulated genes		
Gene	Log_2_FC	*p* value
SPINK1	4.41974	2.47*E*-88
SPP1	3.73200	8.14*E*-131
COL10A1	3.18065	7.47*E*-118
EEF1A2	2.98860	3.87*E*-59
CRABP2	2.97369	6.13*E*-88
GCNT3	2.87337	5.06*E*-68
MMP12	2.85476	6.44*E*-69
MMP1	2.81899	2.73*E*-55
MMP11	2.70584	1.42*E*-66
TMPRSS4	2.65579	1.24*E*-116
COL1A1	2.62413	1.70*E*-88
TOP2A	2.57396	3.07*E*-94
CST1	2.57367	1.52*E*-57
CEACAM5	2.55582	2.61*E*-52
CDH3	2.55139	5.29*E*-86
HMGB3	2.53340	7.97*E*-110
S100P	2.51783	6.32*E*-34
PCP4	2.50192	5.64*E*-54
COMP	2.48062	1.14*E*-56
ITPKA	2.47468	1.30*E*-53
CXCL13	2.44554	3.10*E*-48
NMU	2.43325	1.78*E*-74
MMP9	2.42631	3.87*E*-86
TK1	2.40426	7.22*E*-81
UBE2C	2.38795	1.61*E*-102
LGSN	2.34192	5.47*E*-66
CDC20	2.32945	3.90*E*-72
MELK	2.27942	3.28*E*-106
TPX2	2.24109	3.23*E*-111
BIRC5	2.19958	9.24*E*-79
CP	2.16965	2.78*E*-44
CXCL14	2.08959	9.80*E*-42
HJURP	2.08757	1.04*E*-99
CRLF1	2.08375	3.08*E*-35
PITX1	2.07558	2.05*E*-38
SFN	2.06306	1.89*E*-53
CCNB1	2.06059	1.35*E*-100
GREM1	2.05827	3.69*E*-62
CENPF	2.04220	9.45*E*-110
PHLDA2	2.03621	6.31*E*-63

**(b):** 

Downregulated genes		
Gene	Log_2_FC	*p* value
CA4	-4.46829	4.47*E*-199
TMEM100	-4.46722	1.04*E*-163
FABP4	-4.44449	1.28*E*-204
AGER	-4.42758	1.32*E*-187
FAM107A	-4.27770	1.13*E*-199
FCN3	-4.11945	5.98*E*-158
HBA2	-3.79644	2.07*E*-159
SCGB1A1	-3.77162	3.30*E*-58
CLDN18	-3.76781	5.36*E*-131
SOSTDC1	-3.71859	2.61*E*-143
SLC6A4	-3.71846	3.31*E*-138
SFTPC	-3.67943	5.85*E*-77
ADH1B	-3.64640	1.03*E*-147
HBB	-3.64609	4.82*E*-87
CYP4B1	-3.61022	6.84*E*-97
WIF1	-3.60729	1.09*E*-64
CAV1	-3.58362	9.24*E*-148
TCF21	-3.51528	7.17*E*-237
FHL1	-3.38016	1.55*E*-152
TNNC1	-3.37549	4.89*E*-157
PGC	-3.36845	1.21*E*-60
LYVE1	-3.31830	1.19*E*-202
MFAP4	-3.24097	1.82*E*-125
FMO2	-3.23925	5.63*E*-181
CPB2	-3.22462	1.35*E*-89
SDPR	-3.17437	1.15*E*-122
FOSB	-3.15933	1.39*E*-65
MT1M	-3.05645	1.24*E*-98
TGFBR3	-3.03319	6.31*E*-117
VIPR1	-2.97732	8.36*E*-112
PLA2G1B	-2.95468	2.22*E*-97
HIGD1B	-2.93753	3.31*E*-166
RAMP3	-2.93655	4.44*E*-131
CDH5	-2.92926	6.30*E*-146
TSPAN7	-2.91740	4.02*E*-107
GDF10	-2.88654	2.28*E*-148
CALCRL	-2.86974	1.15*E*-145
BCHE	-2.80317	2.48*E*-145
FOXF1	-2.77773	1.35*E*-136
RAMP2	-2.77632	7.01*E*-121

**Table 4:** Cooccurrence or mutual exclusive alterations of 7 hub genes in LUAD ranked by *p* value.

Gene A	Gene B	*p* value	Log odds ratio	Association
CDH5	PECAM1	<0.001	>3	Tendency towards cooccurrence
CDH5	VWF	<0.001	>3	Tendency towards cooccurrence
VWF	PECAM1	0.007	>3	Tendency towards cooccurrence
MMP9	SPP1	0.097	2.315	Tendency towards cooccurrence

The correlation analysis of 7 hub genes showed that the relationships of gene pairs that were statistically significant (*p* < 0.05) all had a tendency towards cooccurrence. Log odds ratio > 0: association towards cooccurrence. Log odds ratio ≤ 0: association towards mutual exclusivity. *p* value < 0.05: significant association. *p* value: derived from Fisher Exact Test. Log odds ratio: quantifies how strongly the presence or absence of alterations in gene A is associated with the presence or absence of alterations in gene B in the selected tumors.

## Data Availability

The datasets presented in this study can be found in online repositories. The names of the repository/repositories and accession number(s) can be found in the article. The raw data supporting the conclusions of this manuscript will be made available by the authors, without undue reservation, to any qualified researcher.

## Abbreviations

CDH5: Cadherin-5
DEGs: Differentially expressed genes
EMT: Epithelial-to-mesenchymal transition
GEO: Gene Expression Omnibus
KM Plotter: Kaplan-Meier Plotter
LC: Lung cancer
LUAD: Lung adenocarcinoma
Log_2_FC: Log_2_ fold change/logarithm of fold change
MET: Mesenchymal-to-epithelial transition
NSCLC: Non-small-cell lung carcinoma
PECAM1: Platelet endothelial cell adhesion molecule 1
PPI: Protein-protein interaction
SCLC: Small cell lung carcinoma
VWF: von Willebrand factor.
